# Validation Study on Automated Sleep Stage Scoring Using a Deep Learning Algorithm †

**DOI:** 10.3390/medicina58060779

**Published:** 2022-06-09

**Authors:** Jae Hoon Cho, Ji Ho Choi, Ji Eun Moon, Young Jun Lee, Ho Dong Lee, Tae Kyoung Ha

**Affiliations:** 1 Department of Otorhinolaryngology-Head and Neck Surgery, Konkuk University School of Medicine, 120-1, Neungdong-ro, Gwangjin-gu, Seoul 05030, Korea; jaehoon@kuh.ac.kr; 2 Department of Otorhinolaryngology-Head and Neck Surgery, Soonchunhyang University College of Medicine, Bucheon Hospital, 170, Jomaru-ro, Bucheon 14584, Korea; 3 Department of Biostatistics, Clinical Trial Center, Soonchunhyang University Bucheon Hospital, 170, Jomaru-ro, Bucheon 14584, Korea; moon6188@schmc.ac.kr; 4 Honeynaps Research and Development Center, Honeynaps Co., Ltd., 4F, 529, Nonhyeon-ro, Gangnam-gu, Seoul 06126, Korea; tony.lee@honeynaps.com (Y.J.L.); bosco.yi@honeynaps.com (H.D.L.); sean.ha@honeynaps.com (T.K.H.)

**Keywords:** polysomnography, sleep stages, deep learning, algorithms

## Abstract

*Background and Objectives:* Polysomnography is manually scored by sleep experts. However, manual scoring is a time-consuming and labor-intensive task. The goal of this study was to verify the accuracy of automated sleep-stage scoring based on a deep learning algorithm compared to manual sleep-stage scoring. *Materials and Methods:* A total of 602 polysomnography datasets from subjects (Male:Female = 397:205) aged 19 to 65 years (mean age, 43.8, standard deviation = 12.2) were included in the study. The performance of the proposed model was evaluated based on kappa value and bootstrapped point-estimate of median percent agreement with a 95% bootstrap confidence interval and R = 1000. The proposed model was trained using 482 datasets and validated using 48 datasets. For testing, 72 datasets were selected randomly. *Results:* The proposed model exhibited good concordance rates with manual scoring for stages W (94%), N1 (83.9%), N2 (89%), N3 (92%), and R (93%). The average kappa value was 0.84. For the bootstrap method, high overall agreement between the automated deep learning algorithm and manual scoring was observed in stages W (98%), N1 (94%), N2 (92%), N3 (99%), and R (98%) and total (96%). *Conclusions:* Automated sleep-stage scoring using the proposed model may be a reliable method for sleep-stage classification.

## 1. Introduction

Sleep accounts for one-third of a typical human lifetime and plays a very important role in maintaining good health. It is widely known that adequate sleep is associated with mental function and health, including the improvement of attention, learning, cognition, and memory [1,2]. Additionally, appropriate sleep is also related to the restoration of physical health [1]. Abnormal sleep duration or poor sleep quality caused by sleep disorders can result in cardiovascular, metabolic, neurological, and malignant diseases [3,4].

A sleep test or polysomnography is generally recommended for identifying sleep status and detecting sleep diseases, such as sleep-related breathing disorders, parasomnia, and sleep-related movement disorders [5]. Polysomnography is divided into four levels according to the setting (e.g., attended or unattended and in-laboratory or in-home) and number/type of leads (e.g., electroencephalography (EEG), electrooculography (EOG), electrocardiography, chin and limb electromyography (EMG), nose and mouth airflow, chest and abdomen respiratory effort, and oximetry) [6].

Attended overnight full-channel (≥7), in-laboratory polysomnography (level 1) is the most accurate and reliable type of sleep test. However, it has disadvantages, such as sleeping overnight in a laboratory, high cost, “first-night” effects, and inconvenience [7]. One limitation of polysomnography is that numerous raw data from overnight testing must be manually scored by sleep experts over several hours. In particular, the scoring of sleep stages based on EEG, EOG, and EMG data is a detailed, time-consuming, difficult, and labor-intensive task. Additionally, scoring outcomes may differ slightly depending on the scorer [8,9].

To overcome these drawbacks, various automated or computer-aided sleep staging algorithms have been developed and tested [10,11,12,13,14,15,16,17,18]. Results have been reported with various ranges of accuracy and agreement according to different materials and/or methods (e.g., dataset, sample size, classifier, and signal type and number). Regardless, ongoing efforts to develop more accurate and reliable algorithms for automated or computer-aided sleep-stage scoring are necessary. The goal of this study was to assess the precision of a newly developed automated sleep-staging algorithm based on comparisons between automated sleep-stage scoring using the developed deep learning algorithm and manual sleep-stage scoring.

## 2. Materials and Methods

### 2.1. Ethical Statement and Study Population

The present study was designed as a retrospective analysis of polysomnographic data collected by the Soonchunhyang University Bucheon Hospital. This study was approved by the Institutional Review Board of Soonchunhyang University Bucheon Hospital, and informed consent was waived (SCHBC 2018-12-007). All methods of our study were performed in accordance with the relevant guidelines and regulations, including the Declaration of Helsinki. We included 602 datasets from subjects (Male:Female = 397:205) aged 19 to 65 years (mean age, 43.8, standard deviation [SD] = 12.2). About two-thirds (*n* = 397) of this dataset consists of subjects diagnosed with obstructive sleep apnea (mild type = 136, moderate type = 100, and severe type = 161), and mean AHI was 22.9 ± 26.0 events / hour of total sleep time. The percentage of total sleep time in each sleep stage W, N1, N2, N3, and R was 16.5 ± 13.7, 15.2 ± 11.2, 50.2 ± 13.5, 2.7 ± 4.7, and 15.4 ± 6.3, respectively. The datasets were collected using polysomnography at a tertiary university hospital between November 2016 and January 2019. Data were excluded from the dataset when data analysis was not possible, such as when the data were flawed or missing.

### 2.2. Dataset

Personal identification information was removed from the data, and the data were exported in the European data format. All polysomnographic data were acquired and scored by experienced technologists according to the American Academy of Sleep Medicine (AASM) scoring manual (Version 2.3) [19]. Six-channel EEG, two-channel EOG, and one-channel chin EMG were used for analysis. Approximately 75% of polysomnographic data represented patients with sleep disorders, and approximately 25% of the polysomnographic data represented normal subjects.

### 2.3. Feature Extraction

Time- and frequency-domain features, namely line length, kurtosis, and spectrograms of EEG signals, were extracted from 30 s epoch bases. Frequency-domain features were defined as delta-band power spectrum density (PSD), theta-band PSD, alpha-band PSD, and beta-band PSD using a multi-taper spectral analysis method with 29 sub-epochs with lengths of 2 s and overlaps of 1 s to capture the influences of sleep spindle and K-complex signals [14]. All PSDs were divided by the total power of an epoch, and the PSDs from contralateral channels (F3-M2 and F4-M1, C3-M2 and C4-M1, and O1-M2 and O2-M1) were averaged to reduce noise. Additionally, we used delta-theta, theta-alpha, and delta-alpha power ratios as features. The 95% values, minimum values, mean values, and standard deviations of these features were also used. Energy-constant band of power spectra was used as the features of EOG signals, and energy signals were used as the features of EMG signals [18].

### 2.4. Performance Evaluation Metrics

#### 2.4.1. Kappa Value

The kappa value was used as a performance evaluation metric for our proposed model. Kappa is a statistical index to use to measure inter-rater reliability for categorical items [20]. Because kappa considers the possibility of the agreement by chance, it represents a more robust statistical metric than percent agreement. In this study, we used Cohen’s kappa to evaluate the agreement of each sleep stage.

#### 2.4.2. Bootstrap Method (Bootstrapped Point-Estimate of Median Percent Agreement with a 95% Bootstrap Confidence Interval [CI])

In the machine learning field, the bootstrapping method is widely used to estimate the performance of machine learning algorithms. This method provides summary statistics, such as means and standard deviations, to algorithm developers. To obtain these statistics, a resampling technique is applied by sampling a dataset with replacement. The main advantage of this method is that the performance of an estimator can be presented with CIs. This feature is not available with other methods. The basic principle of this method is that inferences regarding a population can be estimated by resampling sample data [21]. With an unknown population, one cannot know the true error in a sample statistic. However, if the population consists of sampled data, which are known, the quality of inference of true samples based on resampled data is measurable. Therefore, the bootstrap resampling method is used for estimating the performance of machine learning algorithms when making predictions for data that are not included in the training data. The resulting metrics are CIs for positive, negative, and overall agreement.

### 2.5. System Architecture

**Figure 1:**
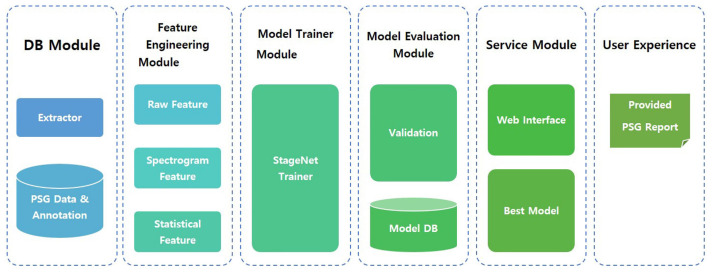
Block diagram of the system architecture. DB: database, PSG: polysomnography.

The proposed model, named StageNet (one of functions in SOMNUM, manufactured by Honeynaps Co. Ltd., Seoul, Korea, configured to distinguish sleep stages), can be divided into six modules. The overall system architecture is presented in Figure 1. The DB module saves EEG, EOG, and EMG data. The feature engineering module extracts predefined features. These extracted features are used for model training. The training results are saved, validated, and tested. The best model is selected by the service module according to the training results. This model is then serviced by user interface software. The final output of StageNet is a sequence of sleep stages with 30 s epochs. A convolutional neural network (CNN), recurrent neural network (RNN), or CNN + RNN can be used as a classifier. In this study, the CNN + RNN exhibited the best agreement with expert scoring results.

### 2.6. Statistical Analysis

Cohen’s kappa statistics were used to quantity the level of agreement between sleep expert scores and the outputs of StageNet based on Soonchunhyang University Bucheon Hospital data (poor agreement: κ < 0, slight agreement: κ = 0.01–0.20, fair agreement: κ = 0.02–0.40, moderate agreement: κ = 0.04–0.60, substantial agreement: κ = 0.06–0.80, almost perfect agreement: κ = 0.08–1.00).

We used 1000 bootstrapped samples to calculate the median percent agreement with a 95% CI. All analyses were conducted using the R software (version 3.4.2; The R Foundation for Statistical Computing, Vienna, Austria; https://www.rproject.org/, accessed on 20 March 2020).

## 3. Results

In this study, confusion matrices, kappa values, and a bootstrap method (bootstrapped point-estimate of median percent agreement with a 95% percentile bootstrap CI with R = 1000) were used to evaluate the performance of the proposed algorithm. We used 482 datasets for training, 48 datasets for validation, and 72 randomly selected datasets for testing.

### 3.1. Confusion Matrix

A confusion matrix for the proposed model is presented in Table 1. One can see relatively high concordance rates between the proposed model and manual scoring for stages W (94%), N1 (83.9%), N2 (89%), N3 (92%), and R (93%).

### 3.2. Kappa Values

**Figure 2:**
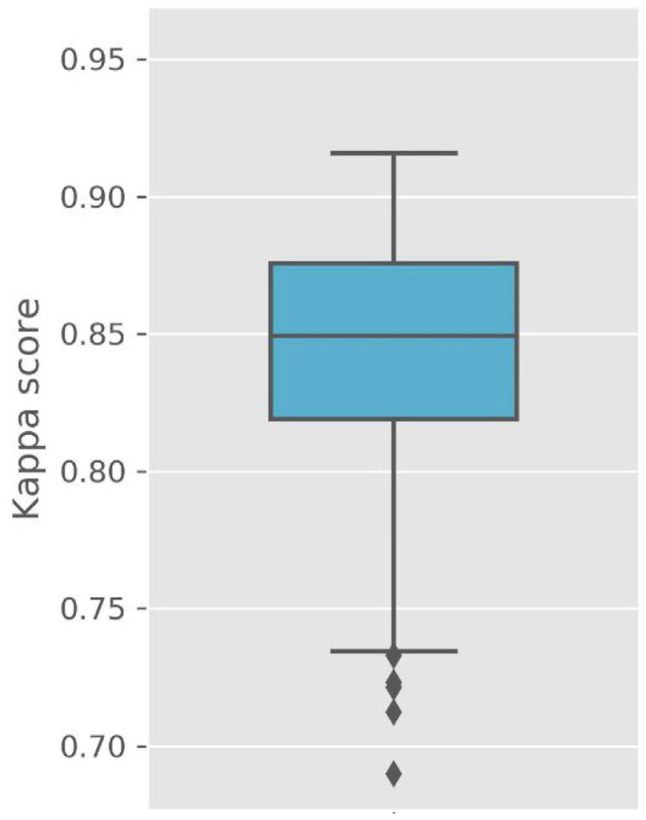
Cohen’s kappa value. Kappa value: median of 0.85, average of 0.84, standard deviation of 0.05, maximum value of 0.92, and minimum value of 0.69.

Figure 2 presents Cohen’s kappa values for the testing dataset. The kappa values represent excellent outcomes (kappa values: median of 0.85, average of 0.84, SD of 0.05, maximum value of 0.92, minimum value of 0.69). However, these values simply indicate agreement between sleep expert scores and the outputs of StageNet. Therefore, we applied a bootstrapping method to estimate accuracies and CIs for polysomnographic data not used for model training.

### 3.3. Bootstrapping Method

The estimation results of the bootstrapping method are presented in Table 2. A group of 72 data samples were selected to measure the CIs of positive, negative, and overall agreement between sleep stages.

High overall agreement between the proposed model and manual scoring can be observed for stages W (98%), N1 (94%), N2 (92%), N3 (99%), and R (98%). The average overall agreement between the two scoring methods for these sleep stages is 96%.

**Figure 3:**
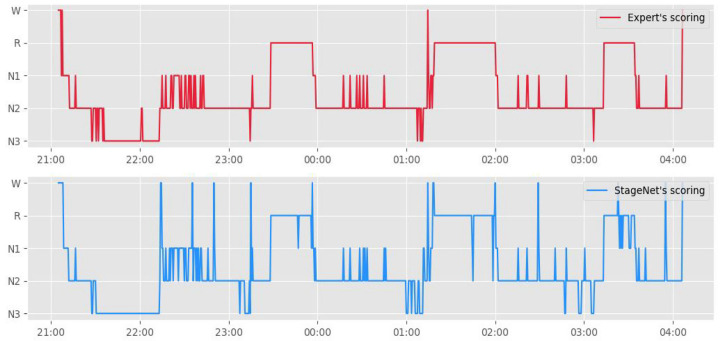
Hypnogram comparison between manual sleep-stage scoring and automated sleep-stage scoring based on a deep learning algorithm (StageNet).

Figure 3 presents a hypnogram of the model outputs and human scorer outputs. The blue line represents the automated sleep staging algorithm, and the orange line represents sleep experts.

## 4. Discussion

The results of our experiments revealed good concordance rates and high overall agreement between manual sleep-stage scoring and automated sleep-stage scoring based on StageNet.

Even though a standard for sleep-stage scoring was provided by the AASM, the scoring results of sleep technicians may differ based on ambiguity in brain waves and human errors. Danker-Hopfe et al. [8] investigated the inter-rater reliability of sleep-stage scoring according to the AASM and Rechtschaffen and Kales (R&K) standards. They calculated kappa values of 0.76 (overall agreement = 82%) for the AASM standard and 0.68 (overall agreement = 80.6 %) for the R&K standard (*n* = 72) [4]. Deng et al. [9] assessed the inter-center reliability (Chinese center (five doctors) versus American center (two doctors)) of sleep-stage scoring according to the AASM manual (2014). Their results revealed a kappa value of 0.75 and an overall agreement of 82.1% (*n* = 40). In this study, we also used the kappa value as an evaluation index to verify the performance of a newly developed automated sleep-stage scoring algorithm and observed a kappa value of 0.85. Compared to earlier validation studies on automated sleep-stage scoring, the results of this study revealed excellent outcomes. Stepnowsky et al. [10] analyzed the agreement between an automated sleep-stage scoring algorithm and manual sleep-stage scoring based on polysomnographic results for 44 patients. The kappa values for automated versus manual scoring (three raters) ranged from 0.42 to 0.63, and the overall agreement ranged from 57.1% to 72.6%. Hassan and Bhuiyan [11] evaluated the accuracy of computer-aided sleep-stage scoring based on spectral features in the tunable-Q-factor wavelet transform domain and calculated a kappa value of 0.86 and overall accuracy of 72.3% based on the AASM standard for five stage-sleep classifications (*n* = 20). Zhang et al. [12] estimated the precision and agreement of automated sleep-stage scoring using a deep learning algorithm based on the Sleep Heart Health Study (*n* = 5213). They calculated a kappa value of 0.82.

Various classifiers based on automated or computer-aided sleep-staging algorithms have been developed (e.g., fuzzy algorithms, linear discriminant analysis, random forests, extreme learning machines, conditional random fields with time information, decision trees, and deep learning) [10,11,12,13,14,15,16,17,18]. Recently, validation studies on automated sleep-stage scoring based on deep learning algorithms have shown increased kappa values compared to other types of classifiers [12,17]. In this study, we used an automated sleep-stage scoring method based on a deep learning algorithm and found that StageNet achieves excellent performance for automated sleep-stage scoring. These outcomes are intuitive because deep learning algorithms represent nonlinear functions better than other classifiers. Another advantage of deep learning classifiers is performance improvement based on data accumulation. As polysomnographic data are accumulated, they can be used for additional training to improve the performance of deep learning classifiers.

Most validation studies on automated sleep-stage scoring have reported that N1 is the hardest sleep stage to classify among the five sleep stages (W, N1, N2, N3, and R). The results of this study also demonstrate that the concordance rate for stage N1 is lower than those for stages W (94%), N2 (89%), N3 (92%), and R (93%). There are several possible reasons for these results. First, after scoring stage N2, a scorer continues to score the following epochs with low-amplitude, mixed-frequency (LAMF) EEG signals without sleep spindles or K-complexes according to the AASM scoring manual [19]. Therefore, if N2 is scored based on the existence of K-complexes or sleep spindles, and the next epochs all represent LAMF EEG activity until additional events occur, such as arousal signals, then these epochs should be scored as N2. However, it is not easy for automated sleep-staging algorithms to learn or apply these rules. If LAMF signals are dominant over one epoch, that epoch is likely to be scored as stage N1 regardless of the prior scoring of the epoch. Second, following stage R, a scorer reviews previous epochs with low chin EMG tones and without rapid eye movements [19]. Specifically, a scorer scores previous epochs with LAMF EEG signals without sleep spindles, K-complexes, or intervening arousal as stage R. After scoring stage R, epochs previously scored as stage N1 can be changed to stage R based on the rules of the AASM scoring manual. It is also difficult for automated sleep staging algorithms to learn these rules. Third, it is widely known that up to approximately 20% of the general population generates little or no alpha activity [22]. For individuals who generate little or no alpha rhythm, scorers will score theta (4–7 Hz) EEG activity with background frequencies more than 1 Hz lower than stage W as stage N1 [19]. If such individuals are included in a dataset, the automated sleep-staging algorithm can have difficulty scoring stage N1.

This validation study has a few limitations. First, it was designed as a retrospective evaluation of an automated sleep-stage-scoring system. Although this study was conducted using a relatively large dataset compared to other studies, additional polysomnographic data should be accumulated to increase the accuracy of deep learning algorithms for sleep-stage scoring. Second, this study was conducted with subjects aged 19 to 65 years. Future studies on subjects younger than 19 years of age or over 65 years of age are needed. Additionally, our clinical trial was conducted using subjects from a single institute. Therefore, further evaluation of patients at other institutes is required.

## 5. Conclusions

A newly developed automated deep learning algorithm called StageNet exhibited excellent performance and may be a reliable tool for sleep-stage scoring in adults aged 19 to 65 years. However, additional research is required to improve the accuracy of the deep learning algorithm for sleep-stage scoring.

## Figures and Tables

**Table 1:** Confusion matrix.

		Automated Sleep-Stage Scoring (StageNet)				
		W	N1	N2	N3	R
Sleep expert	W	94%	2.4%	2.2%	0.1%	1.3%
	N1	2.8%	83.9%	10.9%	0.1%	2.3%
	N2	1.1%	3.5%	89%	3.7%	2.7%
	N3	0.14%	0.21%	7.1%	92%	0.55%
	R	1.2%	2.1%	3.6%	0.1%	93%

W, wakefulness; N1, non-rapid-eye-movement 1; N2, non-rapid-eye-movement 2; N3, non-rapid-eye-movement 3; R, rapid-eye-movement.

**Table 2:** Bootstrapped point-estimate of median percent agreement (%) with a 95% bootstrap confidence interval.

	Total Epochs (*n* = 72 Subjects, 69,591 Epochs)	R = 1000 Resamples		
		Positive Agreement (PA)	Negative Agreement (NA)	Overall Agreement (OA)
W	14,662	95% (95–95%)	98% (98–99%)	98% (97–98%)
N1	6577	73% (71–74%)	96% (96–96%)	94% (94–94%)
N2	33,319	92% (91–92%)	93% (92–93%)	92% (92–93%)
N3	4548	93% (93–94%)	99% (98–99%)	99% (98–99%)
R	10,485	94% (94–95%)	99% (98–99%)	98% (98–98%)
Total	69,591	90% (90–91%)	97% (96–97%)	96% (96–96%)

W, wakefulness; N1, non-rapid-eye-movement 1; N2, non-rapid-eye-movement 2; N3, non-rapid-eye-movement 3; R, rapid-eye-movement.

## Data Availability

All relevant data are within the manuscript.
